# Luteolin Regulates the Differentiation of Regulatory T Cells and Activates IL-10-Dependent Macrophage Polarization against Acute Lung Injury

**DOI:** 10.1155/2021/8883962

**Published:** 2021-01-18

**Authors:** Ke Xie, Yu-sen Chai, Shi-hui Lin, Fang Xu, Chuan-jiang Wang

**Affiliations:** Department of Critical Care Medicine, The First Affiliated Hospital of Chongqing Medical University, Chongqing, China

## Abstract

**Objectives:**

Inflammatory disease characterized by clinical destructive respiratory disorder is called acute lung injury/acute respiratory distress syndrome (ALI/ARDS). Studies have shown that luteolin exerts anti-inflammatory effects by increasing regulatory T cells (Tregs). In this study, we aimed to determine the effects of luteolin on ALI/ARDS and Treg differentiation.

**Methods:**

In this paper, we used cecal ligation puncture (CLP) to generate an ALI mouse model to determine the effects of luteolin on ALI/ARDS. Lung tissues were stained for interleukin- (IL-) 17A and myeloperoxidase (MPO) by immunohistochemical analysis. The levels of Treg-related cytokines in serum and bronchoalveolar lavage fluid (BALF) of mice were detected. The protein levels of NF-*κ*B p65 in lung tissues were measured. Macrophage phenotypes in lung tissues were measured using immunofluorescence. The proportion of Tregs in splenic mononuclear cells and peripheral blood mononuclear cells (PBMCs) was quantified. Furthermore, *in vitro*, we evaluated the effects of luteolin on Treg differentiation, and the effects of IL-10 immune regulation on macrophage polarization were examined.

**Results:**

Luteolin alleviated lung injury and suppressed uncontrolled inflammation and downregulated IL-17A, MPO, and NF-*κ*B in the lungs of CLP-induced mouse models. At this time, luteolin upregulated the level of IL-10 in serum and BALF and the frequency of CD4^+^CD25^+^FOXP3^+^ Tregs in PBMCs and splenic mononuclear cells of CLP mice. Luteolin treatment decreased the proportion of M1 macrophages and increased the proportion of M2 macrophages in lungs of CLP-induced mouse models. *In vitro*, administration of luteolin significantly induced Treg differentiation, and IL-10 promoted the polarization of M2 macrophages but reduced the polarization of M1 macrophages.

**Conclusions:**

Luteolin alleviated lung injury and suppressed uncontrolled inflammation by inducing the differentiation of CD4^+^CD25^+^FOXP3^+^ Tregs and upregulating the expression of IL-10. Furthermore, the anti-inflammatory cytokine IL-10 promoted polarization of M2 macrophages *in vitro*. Luteolin-induced Treg differentiation from naïve CD4^+^ T cells may be a potential mechanism for regulating IL-10 production.

## 1. Introduction

Acute respiratory distress syndrome (ARDS) is a clinically devastating respiratory disorder [1, 2]. Even though clinical management and treatment have improved recently, the mortality rate of severe ARDS remains approximately 46.0% [3]. One of the most promising pharmacological approaches for treating ARDS is based on the pathophysiology of ARDS, i.e., the injury of alveolar epithelial and endothelial cells. However, studies have shown that molecular mechanisms underlying the inflammatory responses are critical to the development of ARDS. In the control and treatment of diseases, it is necessary to adjust and control immune cells and lung microenvironment in a timely manner [4]. Current research studies focus on the identification of drugs for the treatment of ARDS. Personalized pharmacologic therapy for ARDS is one of the most promising ways to treat this disorder [5].

Luteolin (3,4,5,7-tetrahydroxyflavone) can be extracted from many medicinal plants and some common vegetables and fruits, including broccoli, onion leaves, carrot, pepper, cabbage, and apple [6, 7]. It possesses numerous beneficial medicinal properties, such as antioxidative, anti-inflammatory, cardioprotective, neuroprotective, and anti-allergic [8, 9]. A previous study on acute lung injury (ALI) caused by sepsis has shown that luteolin has protective therapeutic effects [10]. Regulatory T cells (Tregs) are special T cells that have anti-inflammatory and antiapoptotic properties. These cells can reduce inflammation-induced tissue damage and create an appropriate immune microenvironment via multiple mechanisms. In ARDS, Tregs function as a metronome for “inflammation factor storm” regulation [4]. They can serve as targets for the treatment of ARDS. Tregs are generated by the differentiation of naïve CD4^+^ T cells. These cells exert their immunoregulatory effects by affecting macrophage polarization through interleukin-10 (IL-10) [11, 12]. Interestingly, luteolin alleviates airway inflammation mainly because it promotes the differentiation of CD4^+^CD25^−^ T cells into CD4^+^CD25^+^ Tregs [13]. In the present study, we investigated whether luteolin exerts a therapeutic effect through regulation of Tregs and IL-10 immune modulation in ARDS.

To validate our hypothesis, we establish a cecal ligation and puncture- (CLP-) induced ALI mouse model and measured the proportion of Tregs in splenic mononuclear cells and peripheral blood mononuclear cells (PBMCs), and identified the polarization of macrophages in lung tissue. Furthermore, *in vitro*, we evaluated the effects of luteolin on Treg differentiation and the effects of IL-10 immune regulation on macrophage polarization.

## 2. Materials and Methods

### 2.1. Animals

All the mice in this experiment were male C57BL/6 mice aged 8–12 weeks and were purchased from the Experimental Animal Center of Chongqing Medical University. Before the experiment, the mice were acclimatized to a room temperature of 22°C. While adjusting the light/dark cycle to 12 h/12 h, the mice were provided food and water *ad libitum* for seven days. This study was conducted in accordance with the recommendations of the Ethics Committee of the First Affiliated Hospital of Chongqing Medical University. The protocol was approved by the Ethics Committee of the First Affiliated Hospital of Chongqing Medical University. The number is Lot 2016-34.

### 2.2. CLP-Induced Model

CLP was performed under anesthesia to determine the position of the cecum. A 3-0 silk suture at 75% of the distance from the tip was made with a 21-gauge needle. Sham-operated control mice were subjected to the same surgical laparotomy; the cecum was exteriorized and manipulated as described, but was not ligated or punctured. After 24 h, the experimental mice in the sham operation or CLP operation were sacrificed at the same time, and their lung tissues, blood samples, and spleens were collected for analysis. After ligating the blood vessels leading to the lung and left bronchus in the mice, we injected 200 *μ*L phosphate-buffered saline (PBS) into the right lung of the mice through the trachea. After 10 s, we took out the PBS we injected and collected bronchoalveolar lavage fluid (BALF). After two washes, the collected BALF reached 400 cubic feet. Then, we analyzed the collected serum, BALF, spleen, and lung tissues.

### 2.3. Treatment with Luteolin

To clarify the therapeutic effects of luteolin, the mice were intraperitoneally injected with 10% DMSO diluted in PBS (control and CLP groups) or 0.2 mg/kg luteolin (RHAWN™, China) (CLP+Lut group) 1 h after CLP stimulation to assess the role of luteolin in ALI/ARDS. The dosage of luteolin was described in previous studies [14, 15]. Blood, BALF, spleen, and lung tissues were collected at predetermined time points.

### 2.4. Blockade of Tregs *In Vivo*

The anti-mouse CD25/IL-2R*α* antibody (AF2438, R&D Systems, Minnesota, USA) was used to inhibit the production of Tregs in mice. Anti-CD25 antibody (AF2438, R&D Systems) or control IgG1 was administered at a dose of 20 *μ*g/mouse 1 h prior to initiating CLP [16].

### 2.5. Histopathology

The upper lobe of the right lung was treated with 10% formalin PBS and, after fixation for 24 h, was dehydrated across an ethanol gradient and embedded with paraffin wax. Paraffin sections were observed by hematoxylin and eosin (H&E) staining. Lung injury scores were estimated by the Mikawa method [17], which comprises the following four categories of lung injury: alveolar congestion, hemorrhage, gap or vascular wall neutrophil infiltration or aggregation, and alveolar septal thickening or transparent membrane formation, and the score interval of each area was 0–4. No or very slight damage was 0 partitions, mild injury was 1 compartment, moderate injury was between 2 partitions, serious injury was a 3-point interval, and very serious damage was between 4 partitions. As the pathological score of ALI, the higher the total score of accumulated lesions, the more serious the injury.

### 2.6. Western Blot Analysis

Lung tissues were lysed and homogenized in RIPA buffer in the presence of a protease inhibitor and phosphatase inhibitor; nucleoproteins were extracted after cytosolic proteins were isolated. Protein concentrations were determined using a BCA Assay kit (Beyotime, China) according to the manufacturer's instructions. Equal amounts of proteins were separated using SDS-PAGE. The proteins were then transferred to polyvinylidene fluoride (PVDF) membranes, then incubated with anti-phospho-p65 (1 : 500 dilution), anti-p65 (1 : 500 dilution), anti-FOXP3 (1 : 500 dilution), or anti-histone H3 (1 : 1,000 dilution) at 4°C overnight. Densitometric analysis of bands was performed using an imaging system (Bio-Rad). All experiments were repeated thrice using different samples.

### 2.7. Immunohistochemistry

After dewaxing the lung slices with xylene, the lung sections were rehydrated with an alcohol gradient, and then 3% H_2_O_2_ was used to block endogenous peroxidase activity for 10 min. Fifteen minutes later, 0.3% Triton X-100 (50 ul) was added to permeabilize the cells. Then, the slices were incubated in normal goat serum. In a humid environment at 4°C, polyclonal goat anti-mouse IL-17A or polyclonal goat anti-MPO (diluted at 15 *μ*g/mL) was added to the section and incubated overnight. Diaminobenzidine (DAB) was used to visualize IL-17A or MPO, while hematoxylin was used to stain the nuclei. Images were captured using a LEICA CTR 5500 microscope and analyzed using Image-Pro Plus 6.0 software.

### 2.8. Immunofluorescence

0.3% Triton X-100 (50 ul) was added to the frozen lung slices recovered by PBS, and the slices were permeabilized for 15 min. After that, antigen retrieval was performed with sodium citrate solution, and then the slices were incubated in normal goat serum. Polyclonal rabbit anti-mouse F4/80, polyclonal rabbit anti-mouse iNOS, and polyclonal rabbit anti-mouse CD206 were added to the slices in a humidified chamber at 4°C and incubated overnight. Sections were subsequently kept at room temperature for 1 h. Then, at normal room temperature, goat anti-rabbit PE-Cy3 (F4/80), goat anti-rabbit Alexa Fluor (iNOS), and goat anti-rabbit FITC (CD206) were used to stain the section, and nuclei was stained with 4′,6-diamino-2-benzoindole (DAPI) for 1 h. Then, the slices were sealed with neutral glue. Five positive cells at 400x magnification were captured and analyzed. The reagents used in this study were purchased from Servicebio, Wuhan, China.

### 2.9. Mouse Serum and BALF Cytokine Measurements

Serum and BALF were frozen at −80°C until the analysis. TNF-*α* IL-1, IL-6, IL-10, and IL-17A expression levels were determined using a mouse cytokine/chemokine magnetic bead panel kit (eBioscience, San Diego, CA, USA).

### 2.10. PBMCs and Splenic Mononuclear Cell Isolation

PBMCs were isolated from mouse heparinized whole blood using a mouse mononuclear cell isolation kit (TBD Science, Tianjin, China) according to the manufacturer's instructions. The splenic mononuclear cells were isolated by density centrifugation as previously described [18]. Briefly, splenic mononuclear cells were isolated from splenic cell suspensions using Ficoll-Paque density gradient (GE Healthcare, USA). The frequency of CD4^+^CD25^+^FOXP3^+^ Tregs was determined by flow cytometry.

### 2.11. Flow Cytometry

PBMCs and splenic mononuclear cell isolation were isolated from recipient mice and stained for markers using anti-CD4-FITC, anti-CD25-PE, and anti-FOXP3-APC. For intracellular FOXP3 analysis, cells were fixed and permeated using a fixation/permeabilization kit (eBioscience) according to the manufacturer's instructions. At least 10^5^ cells were collected with a CytoFLEX flow cytometer (Beckman Coulter, CA, USA) and were analyzed with FlowJo software V10.

### 2.12. Treg Differentiation

Naïve CD4^+^ T cells were sorted from wild-type C57BL/6 mouse spleens using an EasySep™ Mouse Naïve CD4^+^ T Cell Isolation Kit (STEMCELL). About 500,000 cells were plated in 48-well plates in 0.5 mL of complete RPMI 1640 medium (Gibco) supplemented with 10% FBS (Gibco) and stimulated with anti-mouse CD3 (5 *μ*g/mL, eBioscience) and anti-mouse CD28 (2 *μ*g/mL, eBioscience) plus IL-2 (10 ng/mL, PeproTech), TGF-*β*1 (50 ng/mL, PeproTech), 2 mM L-glutamine (STEMCELL), and 50 mM *β*-mercaptoethanol (Macklin) in the absence or presence of 2 *μ*M luteolin (RHAWN™) for 4 d at 37°C with 5% CO_2_. Finally, CD4-FITC, CD25-PE, and FOXP3-APC were used to analyze the cells.

### 2.13. RAW 264.7 Macrophage Culturing and Treatment

RAW 264.7 macrophages were purchased from the American Type Culture Collection (ATCC, Rockville, MD, USA) and cultured in Dulbecco's modified Eagle's medium (DMEM, Gibco) supplemented with 10% heat-inactivated fetal bovine serum (Gibco). The cells were incubated in a humidified atmosphere at 37°C with 5% CO_2_. The cells were divided into three groups as follows: cells incubated with culture medium containing 0.1% DMSO (control group), cells stimulated with LPS (30 ng/mL; Sigma-Aldrich) (LPS group), and cells stimulated with LPS (30 ng/mL; Sigma-Aldrich) plus IL-10 (100 ng/mL; R&D Systems) (LPS+IL-10 group). After 24 h, the membrane surface molecules were stained with 3 *μ*L/test of PE-conjugated monoclonal anti-mouse CD86 (Invitrogen) or APC-conjugated monoclonal anti-mouse CD206 (Invitrogen) for 30 min at room temperature in the dark according to the manufacturer's instructions.

### 2.14. Statistical Analysis

The analysis of all data is mainly conducted by GraphPad Prism 6.01 (GraphPad software, San Diego, CA, USA) and SPSS 19.0 (IBM, Armonk, NY, USA). All statistical data were expressed as the mean ± standard deviation (SD). To evaluate differences between groups, Student's *t* test, Mann-Whitney *U* test, one- or two-way ANOVA test, and Kruskal-Wallis test were used as appropriate. One-way ANOVA was directly conducted using the least significant difference (LSD) multiple comparison test, Dunn test, or Bonferroni multiple comparison test after special testing. Differences were considered statistically significant at *p* < 0.05.

## 3. Results

### 3.1. Luteolin Inhibits Inflammation and Exerts Therapeutic Effects on ALI

**Figure 1:**
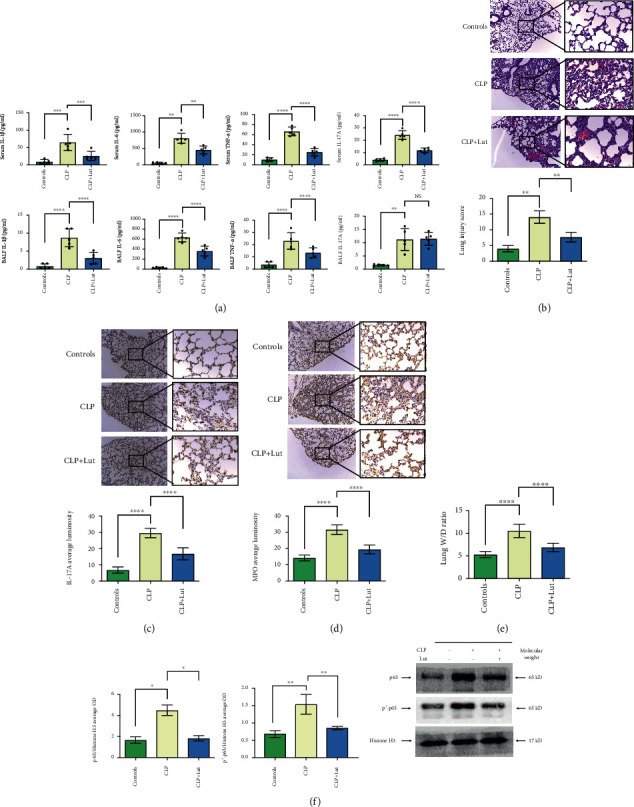
Treatment with luteolin reduces the severity of ALI. (a) Treatment with luteolin reduces the production of IL-1*β*, IL-6, IL-17A, and TNF-*α* in serum and BALF of CLP mice, thereby playing an anti-inflammatory role. IL-1*β*, IL-6, IL-17A, and TNF-*α* levels in serum and BALF of mice were measured using a mouse cytokine/chemokine magnetic bead panel kit. (b) Luteolin treatment alleviates lung injury in CLP-induced ALI mice. Lungs from each experimental group were stained with H&E and processed for histological examination. CLP-induced ALI mice exhibited obvious lung injury. The CLP+Lut group exhibited a significant reduction in the thickness of the alveolar wall, alveolar hemorrhage and collapse, and inflammatory cell infiltration relative to the CLP group. Lung injury in the CLP+Lut group was milder than that in the CLP group. (c, d) Luteolin reduced the proportion of MPO producing neutrophils and IL-17A protein levels in the lungs of CLP mice, thereby playing a protective role in ALI. Immunohistochemical staining for MPO and IL-17A was performed on paraffin-embedded, formalin-fixed lung tissue slices as described in Materials and Methods. (e) Luteolin alleviates pulmonary edema in the CLP-induced mouse model. Pulmonary edema was measured by the lung *W*/*D* weight ratio. (f) Luteolin reduced the nuclear translocation of NF-*κ*B (p65) and NF-*κ*B (p65) phosphorylation activation in lungs of CLP mice. p65 protein levels were measured by western blotting. Each group *n* = 5, experiments are repeatable, and the most representative one was shown. Data of the column graphs are presented as means ± SD. NS: not significant. ^∗^*p* < 0.05, ^∗∗^*p* < 0.01, ^∗∗∗^*p* < 0.001, and ^∗∗∗∗^*p* < 0.0001 by the one-way ANOVA followed by LSD multiple comparison test, compared between the control, CLP, and CLP+Lut groups.

**Figure 2:**
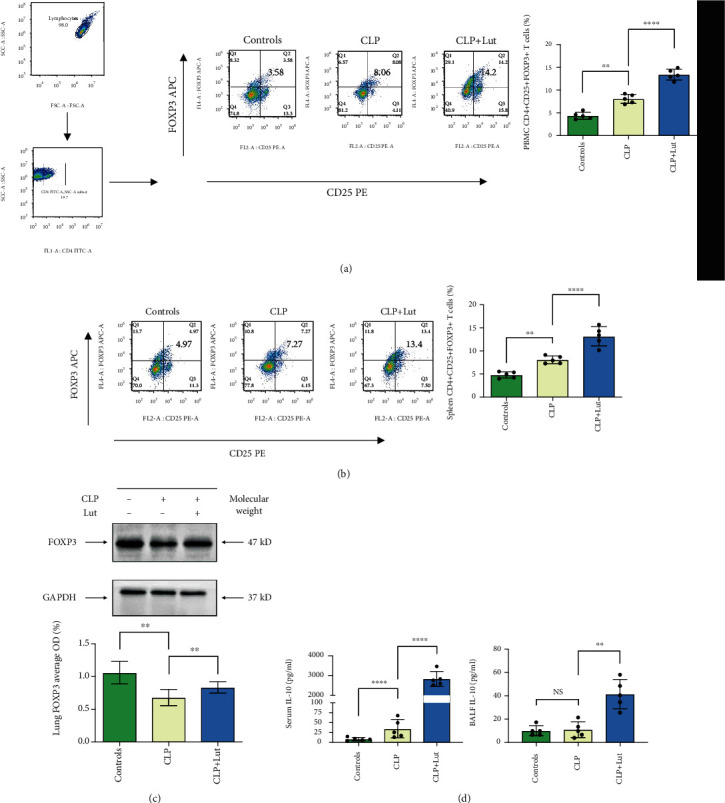
Treatment with luteolin increases the proportion of Tregs and the expression of Foxp3 in the CLP-induced mouse model. (a, b) Treatment with luteolin significantly increased the proportion of CD4^+^CD25^+^FOXP3^+^ Tregs in splenic mononuclear cells and PBMCs of CLP mice. CD4^+^CD25^+^FOXP3^+^ Tregs in splenic mononuclear cells and PBMCs were detected by flow cytometry. (c) Treatment with luteolin increased the expression of Foxp3 in lungs of CLP mice. Foxp3 protein levels were measured by western blot. (d) Treatment with luteolin promotes the expression of IL-10 in serum and BALF of CLP mice, thereby playing an anti-inflammatory role. IL-10 levels in serum and BALF of mice were measured using a mouse cytokine/chemokine magnetic bead panel kit. Each group *n* = 5, experiments are repeatable, and the most representative one was shown. Data of the column graphs are presented as means ± SD. NS: not significant. ^∗^*p* < 0.05, ^∗∗^*p* < 0.01, ^∗∗∗^*p* < 0.001, and ^∗∗∗∗^*p* < 0.0001 by the one-way ANOVA followed by LSD multiple comparison test, compared between the control, CLP, and CLP+Lut groups.

Mice (five per group) were sacrificed 24 h after CLP. Inflammatory tissue damage in the lungs was assessed by histological observation. The results were consistent between experiments; the most representative experimental results are shown in Figure 1. CLP significantly increased lung injury. Treatment with luteolin significantly reduced the extent of lung injury. Comparison of scores of lung injury such as alveolar wall thickening, alveolar hemorrhage, alveolar collapse, and inflammatory cell infiltration in the CLP group revealed that lung injury in the CLP+Lut group was alleviated by luteolin (*p* < 0.01, Figure 1(b)), as shown by the lung injury score. After treatment with luteolin, serum concentrations of cytokines IL-1*β*, IL-6, TNF-*α*, and IL-17A significantly decreased. Moreover, the inflammatory mediators in BALF, including IL-1*β*, IL-6, and TNF-*α*, were also markedly reduced (Figure 1(a)). Serum and BALF IL-10 levels were significantly increased after luteolin treatment (Figure 2(d)).

### 3.2. Effects of Luteolin on the Regulation of IL-17A, Neutrophils, and NF-*κ*B p65 in the Lungs of CLP Mice

After treatment with luteolin, pulmonary IL-17A protein levels decreased (*p* < 0.0001), and the proportion of MPO^+^ neutrophils in the lungs was also reduced (*p* < 0.001) compared with that of the CLP mice (Figures 1(c) and 1(d)). We extracted nuclear proteins from mouse lung tissues to determine the effect of luteolin on NF-*κ*B (p65) nuclear translocation and NF-*κ*B (p65) phosphorylation by western blotting. Compared with the control group, the nuclear translocation of NF-*κ*B (p65) significantly increased after CLP. Meanwhile, the expression level of phosphorylated-p65 (p-p65) in lung tissues of the CLP mice also increased (Figure 1(f)). To assess the degree of lung edema, the *W*/*D* ratio was calculated by dividing the wet weight by the dry weight. Compared with the control, the lung wet/dry weight ratios significantly increased after CLP. After luteolin treatment, lung *W*/*D* weight ratios were reduced (*p* < 0.0001) compared with CLP mice (Figure 1(e)).

### 3.3. Luteolin Treatment Increases the Proportion of CD4^+^CD25^+^FOXP3^+^ Tregs and the Expression of FOXP3 in the CLP-Induced Mouse Model

The proportion of CD4^+^CD25^+^FOXP3^+^ Tregs in splenic mononuclear cells and in PBMCs was detected by flow cytometry. We found luteolin treatment significantly increased the proportion of Tregs in the CLP-induced mouse model (Figures 2(a) and 2(b)). FOXP3 is expressed preferentially in Tregs, and lack of FOXP3 protein leads to lack of functional Tregs. After luteolin treatment, western blot analysis showed that the expression of FOXP3 in lung tissues was upregulated compared with the CLP group (Figure 2(c)).

### 3.4. M1 and M2 Macrophage Phenotypes in the Lungs Are Regulated by Luteolin

**Figure 3:**
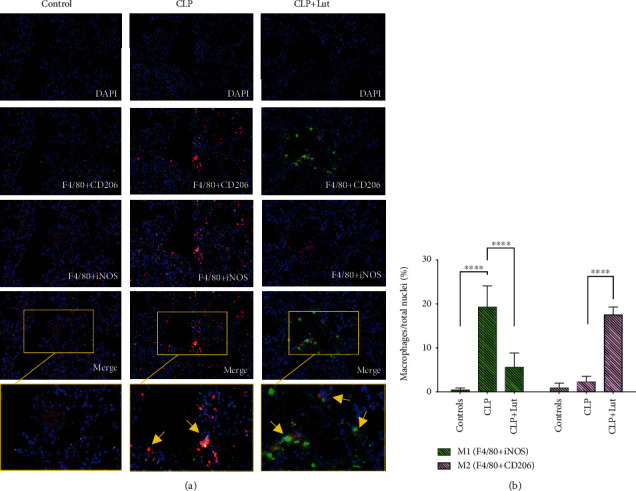
Treatment with luteolin regulates M1 and M2 macrophage phenotypes in lungs of CLP mice. (a) Treatment with luteolin reduces the proportion of M1 macrophages, while increasing the proportion of M2 macrophages in lungs of CLP mice, hence playing a protective role in ALI. The proportion of pink fluorescent iNOS expressing M1 macrophages increased significantly, while lungs of CLP mice were substantially negative for green fluorescent CD206^+^ M2 macrophages. Treatment with luteolin reduced the proportion of M1 while increasing that of M2 macrophages in ALI. Five 400x fields of positive cells were counted and analyzed. Each group *n* = 5, experiments are repeatable, and the most representative one was shown. Data of the column graphs are presented as means ± SD. ^∗∗∗∗^*p* < 0.0001 by the one-way ANOVA followed by LSD multiple comparison test, compared between the control, CLP, and CLP+Lut groups.

Mouse lungs were stained by immunohistochemistry and imaged by fluorescence microscopy. The regulatory levels of luteolin on M1 and M2 macrophages in lung tissues were mainly analyzed by triple immunofluorescence (red: F4/80, pink: iNOS^+^ M1, and green: CD206^+^ M2). Five fields were chosen from each immunohistochemical section; each field was analyzed by microscope (400x). Compared with the CLP group, we found that the number of M1 cells labeled with pink fluorescence after luteolin treatment significantly decreased (*p* < 0.0001), while the number of M2 cells labeled with green fluorescence significantly increased (*p* < 0.0001) (Figure 3).

### 3.5. Protective Effects of Luteolin in ALI Are Related to Tregs and IL-10

**Figure 4:**
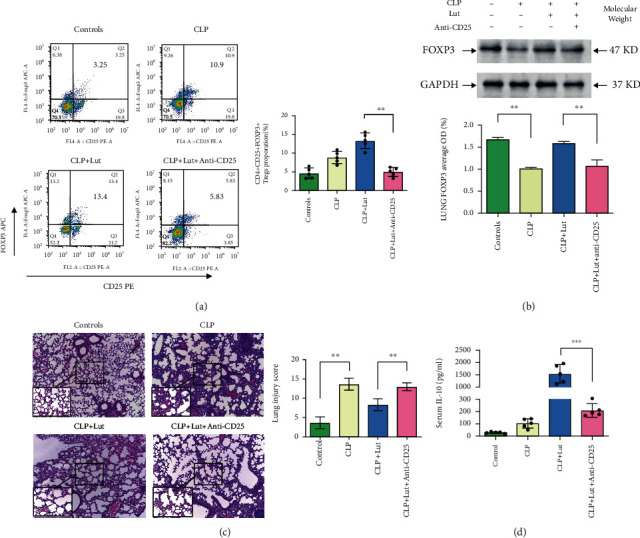
Luteolin plays a protective role in ALI, which is potentially linked to IL-10 and Tregs. (a, c, d) After treatment with CD25 neutralizing antibodies, the number of Tregs in the spleen of mice in the CLP+Lut group was significantly reduced. IL-10 expression was significantly reduced, and lung injury was significantly worse. CD4^+^CD25^+^FOXP3^+^ Tregs in splenic mononuclear cells were detected by flow cytometry. Lungs from each experimental group were stained with H&E and processed for histological examination. Mouse IL-10 serum levels were measured by ELISA. (b) After treatment with CD25 neutralizing antibodies, the expression of Foxp3 in lungs of GL+CLP mice was decreased. Foxp3 protein levels were measured by western blot. Each group *n* = 5, experiments are repeatable, and the most representative one was shown. Data of the column graphs are presented as means ± SD. ^∗∗^*p* < 0.01 and ^∗∗∗^*p* < 0.001 between groups represented by horizontal lines, as calculated by one-way ANOVA followed by LSD multiple comparison test.

To further explore the role of Tregs in CLP-induced lung injury, CD25 neutralizing antibodies were used to deplete the Tregs after luteolin treatment. The results showed that the proportion of Tregs in splenic mononuclear cells and the level of IL-10 in serum decreased after CD25 neutralization. Additionally, lung injury was aggravated. These results suggest that luteolin targets Tregs to regulate IL-10 levels (Figures 4(a), 4(c), and 4(d)). Moreover, after CD25 was neutralized, western blotting showed that the expression of FOXP3 in the lungs decreased compared with that of the CLP+Lut group (Figure 4(b)).

### 3.6. Luteolin Promotes the Differentiation of Naïve CD4^+^ T Cells into Tregs *In Vitro*, and IL-10 Improves the Polarization of M1/M2 Macrophages

**Figure 5:**
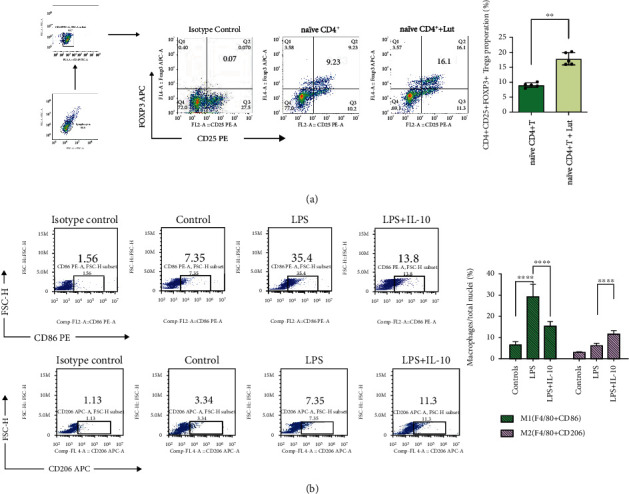
Luteolin promotes the differentiation of naïve CD4^+^ T cells into Tregs, and IL-10 improves the polarization of M1/M2 macrophages. (a) Luteolin significantly promotes the differentiation of naïve CD4^+^ T cells into CD4^+^CD25^+^FOXP3^+^ Tregs. Naïve CD4^+^ T cells were sorted from the spleen of wild-type C57BL/6 mice and cultured as described in Materials and Methods. Tregs were detected by flow cytometry on day 4. (b) IL-10 affects the polarization of M1/M2 macrophages. Proportions of M1/M2 macrophages were examined by flow cytometry on day 3. Each group *n* = 5, experiments are repeatable, and the most representative one was shown. Data of the column graphs are presented as means ± SD. ^∗∗^*p* < 0.01 and ^∗∗∗∗^*p* < 0.0001 comparing the control, LPS, and LPS+IL-10 groups, as calculated by one-way ANOVA followed by the LSD multiple comparison test.

*In vitro*, we tested whether Treg differentiation and regulation of IL-10 secretion were influenced by luteolin. Naïve CD4^+^ T cells were isolated from the mice. Then, these cells were divided into two groups: the control and luteolin groups. The results showed that the proportion of CD4^+^CD25^+^FOXP3^+^ Tregs was significantly higher after treatment with luteolin (Figure 5(a)). In addition, our *in vitro* results show that the anti-inflammatory cytokine IL-10 can promote the polarization of M2 macrophages, while the polarization of M1 macrophages continuously decreased (Figure 5(b)).

## 4. Discussion

ARDS/ALI is a severe diffuse lung disease and is a common cause of death in the intensive care unit [19]. The main feature of ALI is the rupture of the alveolar-capillary interface, the secretion of proinflammatory cytokines, and the infiltration of proinflammatory cells [20]. The regulation of uncontrolled inflammation is important in the development of new treatment strategies for developing lung injury. Tregs regulate the activity of CD4^+^, CD8^+^ T cells, B cells, natural killer cells, dendritic cells, and other immune cells mainly through contact-dependent inhibition or release inhibition of cytokines IL-10 and TGF-*β* and can play an anti-inflammatory role at this time [21]. Tregs play an important role in relieving inflammation and promoting lung repair in ARDS [22, 23]. The mortality of ARDS is closely related to the increase of the CD4^+^CD25^+^FOXP3^+^ Treg ratio in the alveoli [24]. Tregs are considered as potential therapeutic targets for ARDS mainly because they play an important role in alleviating inflammation.

Luteolin is a flavone compound present in many medicinal plants. It not only has anti-inflammatory effects but also has various biological and pharmacological effects [7]. Recent studies have reported that luteolin suppresses the expression of proinflammatory cytokines, including IL-17, IL-1*β*, TNF-*α*, and IL-6, which are key modulators of both acute and chronic inflammation [25]. Luteolin attenuates the effects of sepsis-induced ALI by suppressing the ICAM-1, iNOS pathways, oxidative stress, and NF-*κ*B [15]. In the current study, most research choice pretreatment methods. However, there are an increasing number of experts who think that pretreatments are clinically meaningless. A study comparing different ARDS/ALI models showed that CLP treatment also results in the early elevation of cytokine and plasma TNF-*α* and IL-6 levels 2 h after CLP, and animal body temperature markedly decrease between 2 and 12 h [26]. This is an indicator that systemic inflammation has developed. The development of ARDS/ALI is closely related to uncontrolled inflammation. Treatment within two hours after CLP may aid in developing better therapeutic strategies for the treatment of proinflammatory disorders. In our research, mice in the CLP+Lut group exhibited less severe lung tissue damage compared with those in the CLP group (Figure 1(b)). Protein levels of IL-17A and MPO, which could injure the alveolar epithelium and induce neutrophil aggregation in lung tissue, were significantly reduced (Figures 1(c) and 1(d)). The NF-*κ*B p65 subunit is phosphorylated and translocated into the nucleus to regulate the transcription of proinflammatory mediators [27]. The NF-*κ*B p65 phosphorylation activation and nuclear translocation of NF-*κ*B (p65) significantly increased after CLP. After luteolin treatment, NF-*κ*B p65 phosphorylation activation and nuclear translocation of NF-*κ*B (p65) decreased compared with those of the CLP group (Figure 1(f)). In ARDS, immune cells secrete cytokines and chemokines to initiate and maintain the uncontrolled inflammatory response. The levels of proinflammatory cytokines TNF-*α*, IL-1*β*, IL-6, and IL-17A in mouse serum significantly decreased after treatment with luteolin. Moreover, the inflammatory mediators in BALF, including IL-1*β*, IL-6, and TNF-*α*, were also markedly reduced (Figure 1(a)). In our study, we found that after treatment with luteolin, the concentrations of IL-17A significantly decreased in the mouse serum at 24 h, but did not decrease in mouse BALF. In the process of lung injury, IL-17A expression exhibited differences at various time points; this may be because we used a CLP-induced ALI mouse model. In this model, sepsis originated from a polymicrobial infectious focus within the abdominal cavity, followed by bacterial translocation into the blood compartment, which then triggers a lung inflammatory response [28]. The expression of IL-17A in the mouse serum immediately decreased after inflammation was blocked, but in BALF, this may be delayed. As a consequence, luteolin reduced the degree of lung injury because it can inhibit the production or function of inflammatory mediators.

Currently, information of the mechanisms by which luteolin regulates Tregs is limited. Previous studies have shown that luteolin affects asthma via the induction of FOXP3^+^ and CD4^+^CD25^+^ Tregs or acts as an immunosuppressant mTOR inhibitor to increase the percentage of CD4^+^FOXP3^+^ Tregs posttransplantation [29]. The main results show that (i) the inhibition function of CD4^+^FOXP3^+^ Tregs is enhanced or the proliferation speed of Treg *in vivo* is promoted after activation or (ii) the signal pathway regulated by immunoregulatory cytokines, small molecule inhibitors, or therapeutic antibodies is stimulated [30]. In our study, we observed that the Treg ratio in splenic mononuclear cells and PBMCs of mice significantly increased (Figure 2(a) and 2(b)). CD25, the alpha chain of IL-2 receptor (IL-2R), is expressed in an inducible manner and required for formation of the high affinity IL-2R [31]. CD25 is absent or minimally expressed on resting T and NK cells, but its transcription is potently induced on T cells stimulated via the TCR or IL-2. After T cell activation, CD25 is rapidly induced and high-affinity receptors form [32]. CD25 is an important surface marker for Tregs and is often used to purify and sort Tregs. CD4, CD25, and FOXP3 triple marker-positive is a relative standard method to identify Tregs [33]. Tregs constitutively express CD25, and CD4^+^CD25^+^ Tregs suppress CD4^+^CD25^−^ T cells in a cell contact-dependent manner *in vitro* [34]. In our research, the total number of CD25^+^FOXP3^−^ cells in the flow cytometry plots increased in the CLP+Lut group compared with the CLP group (Figures 2(a) and 2(b)), indicating that luteolin could promote Treg differentiation in ARDS. FOXP3 is specifically in the Tregs expressed and controls their development and function. Western blotting showed that after treatment with luteolin, the expression of FOXP3 in the lungs was also upregulated compared with the CLP group (Figure 2(c)), indicating that luteolin promotes Treg differentiation and the expression of FOXP3 in ARDS. Another study showed that the decrease in FOXP3 expression in patients is due to the decrease of FOXP3 expression in CD4^+^ T cells, which leads to Treg dysfunction in patients [35]. Our study shows that luteolin treatment results in the upregulation of FOXP3 in the lungs compared with CLP mice, which may be related to the increase in lung Tregs and its protective effects. However, the underlying mechanism requires further investigation. Moreover, comparison of the CLP+Lut group with the CLP group revealed that the levels of Treg-related cytokines IL-2 and IL-10 in the CLP+Lut group significantly increased (Figure 2(d)), thus providing preliminary evidence that luteolin regulates Tregs and thereby improves the inflammatory response and reduces lung injury. Simultaneously, Tregs originate from naïve CD4^+^ T cells. A variety of effector T cell lineages, including Tregs, is formed by the differentiation of naïve CD4^+^ T cells. A recent study showed that the inflammatory microenvironment is coupled with metabolism and can induce phenotypic remodeling of CD4^+^ T cells [36]. Thus, it is essential to determine the effects of luteolin. In our *in vitro* study, after stimulation with luteolin, the proportion of CD4^+^CD25^+^FOXP3^+^ Tregs significantly increased (Figure 5(a)). These results indicate that one of the mechanisms by which luteolin regulates Tregs may be by enhancing the differentiation of naïve CD4^+^ T cells into Tregs.

Because Tregs can act as a “cytokine sink” for different effector cytokines [4], the effects of luteolin on Tregs might be mainly mediated by cytokines. One critical suppressive mechanism of Tregs is the secretion of inhibitory cytokines such as IL-10 and TGF-*β* [37]. IL-10 is an anti-inflammatory cytokine and protects against lung injury. In our study, the protective effect of luteolin in ALI is closely related to Tregs and IL-10. The increased expression of IL-10 is also due to luteolin treatment (Figure 2(d)). Luteolin and Tregs have a certain synergistic effect in regulating the expression of IL-10 in cells. However, the protective effect of IL-10 in ALI not only inhibits the production of proinflammatory cytokines but also mediates the activity of neutrophils [38] and dendritic cells [39]. Importantly, IL-10 is a key signaling molecule for macrophages in their response to inflammation [40]. The anti-inflammatory effects of IL-10 are mediated by metabolic reprogramming of macrophages, including inhibition of mTOR signaling and inflammasome activation [11]. Macrophages play an important role in ARDS when they encounter an inflammatory reaction. Macrophages have both proinflammatory and anti-inflammatory effects, which change with the microenvironment of different pathological stages [41]. In terms of macrophage phenotypes in the mouse lung, we found that the M1 phenotype was more prevalent in the CLP group than in the CLP+Lut group, and the opposite was observed for M2 macrophages. Luteolin treatment significantly decreased the proportion of M1 macrophages and increased the proportion of M2 macrophages (Figure 3), which coincides with changes in IL-10 levels in plasma and Tregs of splenic mononuclear cells and PBMCs upon luteolin treatment. A similar observation was reported in a previous study; Il-10 derived from Tregs is one of the main factors affecting macrophage polarization [42]. Therefore, the present study explored the interaction between Tregs and other effector cells (such as macrophages) and the influence of cytokines secreted by Tregs (such as IL-10) on this interaction. Another research also observed that at postinfection, IL-10, an anti-inflammatory cytokine produced by Tregs and antagonizes Th1 cytokines, was also highly increased [43]. In addition, our *in vitro* experiments further proved that IL-10 could induce macrophage polarization toward the M2 phenotype and suppress macrophage polarization toward the M1 phenotype *in vitro* (Figure 5(b)). In lung injury, M1 macrophages play the role of promoter; on the contrary, the level of proinflammatory cytokines in ARDS is limited by M2 macrophage and participates in the process of lung tissue repair. Our experiment suggested that luteolin exerts its anti-inflammatory effects and is closely related to regulating Treg differentiation, promoting IL-10 expression, and then interfering with macrophage polarization.

In sum, luteolin imparts a therapeutic effect on ARDS, which includes the regulation of Tregs and reduction in proinflammatory cytokine production. These therapeutic effects could indirectly affect other immune cells (e.g., macrophages) through cytokines secreted by Tregs. Our findings show that luteolin promotes Treg cell differentiation and increases the production of IL-10, which may interfere with the polarization of macrophages.

## 5. Conclusions

Luteolin alleviates lung injury and suppresses uncontrolled inflammation by inducing the differentiation of CD4^+^CD25^+^FOXP3^+^ Tregs and upregulating the expression of IL-10. Furthermore, the anti-inflammatory cytokine IL-10 can promote polarization of M2 macrophages *in vitro*. Luteolin induces the differentiation of Tregs from naïve CD4^+^ T cells and may be a potential mechanism for regulating IL-10 production.

## Data Availability

All data generated or analyzed during this study are included in this article.
