# Correction to “Luteolin Regulates the Differentiation of Regulatory T Cells and Activates IL‐10‐Dependent Macrophage Polarization against Acute Lung Injury”

**DOI:** 10.1155/jimr/9786427

**Published:** 2025-10-10

K. Xie, Y. Chai, S. Lin, F. Xu, and C. Wang, “Luteolin Regulates the Differentiation of Regulatory T Cells and Activates IL‐10‐Dependent Macrophage Polarization against Acute Lung Injury,” *Journal of Immunology Research* 2021 (2021): 8883962, https://doi.org/10.1155/2021/8883962.

In the article, the authors have identified an error in Figure 1f, whereby the incorrect western blots were used when preparing the figure. The authors have reassessed the correct data and blots, and provided the correct Figure 1f as below:

**Figure 1:**
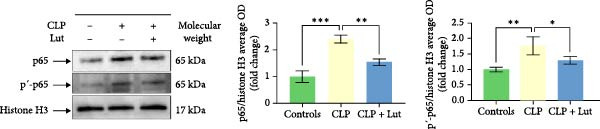
Treatment with luteolin reduces the severity of ALI. (a) Treatment with luteolin reduces the production of IL‐1β, IL‐6, IL‐17A, and TNF‐α in serum and BALF of CLP mice, thereby playing an anti‐inflammatory role. IL‐1β, IL‐6, IL‐17A, and TNF‐α levels in serum and BALF of mice were measured using a mouse cytokine/chemokine magnetic bead panel kit. (b) Luteolin treatment alleviates lung injury in CLP‐induced ALI mice. Lungs from each experimental group were stained with H&E and processed for histological examination. CLP‐induced ALI mice exhibited obvious lung injury. The CLP + Lut group exhibited a significant reduction in the thickness of the alveolar wall, alveolar hemorrhage and collapse, and inflammatory cell infiltration relative to the CLP group. Lung injury in the CLP + Lut group was milder than that in the CLP group. (c, d) Luteolin reduced the proportion of MPO producing neutrophils and IL‐17A protein levels in the lungs of CLP mice, thereby playing a protective role in ALI. Immunohistochemical staining for MPO and IL‐17A was performed on paraffin‐embedded, formalin‐fixed lung tissue slices as described in Materials and Methods. (e) Luteolin alleviates pulmonary edema in the CLP‐induced mouse model. Pulmonary edema was measured by the lung W/D weight ratio. (f) Luteolin reduced the nuclear translocation of NF‐κB (p65) and NF‐κB (p65) phosphorylation activation in lungs of CLP mice. p65 protein levels were measured by western blotting. Each group *n* = 5, experiments are repeatable, and the most representative one was shown. Data of the column graphs are presented as means ± SD. NS: not significant.  ^∗^ *p* < 0.05,  ^∗∗^ *p* < 0.01,  ^∗∗∗^ *p* < 0.001, and  ^∗∗∗∗^ *p* < 0.0001 by the one‐way ANOVA followed by LSD multiple comparison test, compared between the control, CLP, and CLP + Lut groups.

The results and conclusions are not affected by this error.

We apologize for this error.

